# Blood group type A secretors are associated with a higher risk of COVID‐19 cardiovascular disease complications

**DOI:** 10.1002/jha2.180

**Published:** 2021-04-02

**Authors:** Tosti J. Mankelow, Belinda K. Singleton, Pedro L. Moura, Christian J. Stevens‐Hernandez, Nicola M. Cogan, Gyongyver Gyorffy, Sabine Kupzig, Luned Nichols, Claire Asby, Jennifer Pooley, Gabriella Ruffino, Faroakh Hosseini, Fiona Moghaddas, Marie Attwood, Alan Noel, Alex Cooper, David T. Arnold, Fergus Hamilton, Catherine Hyams, Adam Finn, Ashley M. Toye, David J. Anstee

**Affiliations:** ^1^ Bristol Institute for Transfusion Sciences (BITS) NHSBT, Filton Bristol UK; ^2^ NIHR Blood and Transplant Research Unit in Red Cell Products Bristol UK; ^3^ Center for Hematology and Regenerative Medicine Department of Medicine (MedH) Karolinska Institutet Stockholm Sweden; ^4^ School of Biochemistry Biomedical Sciences Building University of Bristol Bristol UK; ^5^ Acute Medical Unit, Southmead Hospital North Bristol NHS Trust Bristol UK; ^6^ Infection Sciences, Southmead Hospital North Bristol NHS Trust Bristol UK; ^7^ Academic Respiratory Unit Southmead Hospital North Bristol NHS Trust Bristol UK; ^8^ Population Health Sciences University of Bristol Bristol UK; ^9^ Bristol Vaccine Centre University of Bristol Bristol UK; ^10^ Cellular and Molecular Medicine Biomedical Sciences Building, University of Bristol Bristol UK

## Abstract

The SARS‐CoV‐2 virus causes COVID‐19, an infection capable of causing severe disease and death but which can also be asymptomatic or oligosymptomatic. We investigated whether ABO blood group or secretor status was associated with COVID‐19 severity. We investigated secretor status because expression of ABO glycans on secreted proteins and non‐erythroid cells are controlled by a fucosyltransferase (FUT2), and inactivating FUT2 mutations result in a non‐secretor phenotype which protects against some viral infections. Data combined from healthcare records and our own laboratory tests (*n* = 275) of hospitalized SARS‐CoV‐2 polymerase chain reaction positive patients confirmed higher than expected numbers of blood group A individuals compared to O (RR = 1.24, CI 95% [1.05, 1.47], *p* = 0.0111). There was also a significant association between group A and COVID‐19‐related cardiovascular complications (RR = 2.56, CI 95% [1.43, 4.55], *p* = 0.0011) which is independent of gender. Molecular analysis revealed that group A non‐secretors are significantly less likely to be hospitalized than secretors. Testing of convalescent plasma donors, among whom the majority displayed COVID‐19 symptoms and only a small minority required hospitalization, group A non‐secretors were slightly over‐represented. Our findings showed that group A non‐secretors are not resistant to infection by SARS‐CoV‐2, but are more likely to experience a less severe form of associated disease.

## 1 INTRODUCTION

Genetic diversity among members of animal species including *Homo sapiens* is essential for their survival in response to newly emergent and evolving pathogens [1]. Human blood group antigens are among the first polymorphic structures encountered by viruses and bacteria upon airborne contact with respiratory, gastrointestinal and urinogenital mucosal surfaces [2]. The carbohydrate antigens of the ABO and Lewis blood group systems are found on mucosal surfaces where their presence is controlled by a fucosyltransferase (FUT2) [3, 4]. In the presence of active *FUT2* A, B, H, and Le^b^ antigens can be expressed on mucosal surfaces. Individuals with this phenotype are known as secretors [5, 6]. In individuals lacking active *FUT2*, known as non‐secretors, only the Le^a^ antigen can be expressed [5]. There are several well studied interactions between host cells and both bacteria (*Helicobacter pylori, Vibrio cholera*) and viruses (noroviruses, rotaviruses) which are known to depend on the presence of these antigens [7, 8]. In particular, it is clearly established that common strains of norovirus and rotavirus fail to infect non‐secretors [9, 10, 11, 12].

A novel coronavirus (severe acute respiratory syndrome coronavirus 2 [SARS‐CoV‐2]) emerged in China in December 2019, causing a pandemic of severe respiratory disease, known as coronavirus disease (COVID‐19) [13]. Currently, more than 112 million people have been infected worldwide, and, of those, more than 2.5 million have died as a result of the disease. It was apparent from the earliest studies that disease severity in individual patients varied considerably, ranging from asymptomatic infection to fatal illness [14]. Zhao et al provided the first evidence of an association between blood group polymorphisms and with disease severity [15]. In a study of over 2000 infected patients from Wuhan, China, it was noted that group A phenotype was found more frequently than expected in patients with COVID‐19, whereas group O occurred less commonly than expected in the general population, suggesting that group A individuals are at greater risk from COVID‐19 than those of group O. This observation led us to undertake a study of the association of blood group polymorphisms (ABO and secretor status) with COVID‐19 severity in hospitalized patients in Bristol, UK.

Here we report a significantly increased frequency of hospitalization for blood group A compared to blood group O patients with COVID‐19. This was accompanied by a significantly higher rate of respiratory failure at admission but had no significant association with length of stay or patient death rate. Importantly, we observe a trend between blood group A and cardiovascular complications, which we confirm in a separate cohort of hospitalized COVID‐19 patients. Investigation of the role of secretor status in relation to disease severity revealed that COVID‐19 incidence in group A non‐secretors was much lower than would be expected if the absence of active *FUT2* had no impact on disease, within the hospitalized patient context. Moreover, this effect was specific to blood group A and was not observed in patients with blood group O.

## 2 METHODS

### 2.1 Epidemiological surveillance

A retrospective cohort analysis of COVID‐19 infected individuals, undertaken as part of an audit on adult patients hospitalized at North Bristol NHS Trust with COVID‐19 infection and was approved by North Bristol NHS Trust Audit and Ethics Committee. Adults admitted from March 27 to July 27, 2020 were identified by searching the Laboratory Information Management System database (Clinisys, WinPath Enterprise). The inclusion criteria were a positive polymerase chain reaction (PCR) result for SARS‐CoV‐2, using the established Public Health England reverse transcriptase PCR (RT‐PCR) assay in use at the time and the requirement for hospitalization. Clinical records were then reviewed to determine patient demographics, preexisting comorbidities, and blood group. Outcomes were assessed 30 days following admission, including the requirement for cardiovascular and respiratory support. A random number generator was used to select 10% of records for review to ensure data accuracy.

### 2.2 Blood samples

Access to and research on healthy donor and COVID‐19 patient or convalescent samples was undertaken using Health Research Authority (HRA) ethical approval, which was reviewed by Leeds West Research Ethics Committee (REC 20/YH/0168). This includes accessing blood samples and clinical outcomes collected by the **DI**agnostic and **S**everity markers of **COV**ID‐19 to **E**nable **R**apid triage (DISCOVER) study of hospitalized COVID‐19 patients and outcomes between April 1 and October 1, 2020. The DISCOVER Study is an observational cohort study of patients with either a PCR positive test or COVID‐19 symptoms at North Bristol NHS Trust. DISCOVER samples were collected under HRA ethical approval, which was reviewed and approved by South Yorkshire Research Ethics Committee (REC No.20/YH/0121). We also accessed 1000 anonymous residual blood samples from NHSBT convalescent plasma donated between May 19 and June 26.

### 2.3 Serology

Red cells were tested serologically for ABO, Rh, and Lewis using relevant DiaClon cards in accordance with the manufacturer's instructions (Bio‐Rad).

### 2.4 Genomic DNA isolation

Genomic DNA was isolated from whole blood samples using the PureLink Genomic DNA Mini kit according to the manufacturer's instructions (Invitrogen).

### 2.5 Genotyping of the *FUT2* G428A polymorphism

ABH antigen secretor status was determined by allele‐specific PCR of the G428A polymorphism in the *FUT2* gene. PCR products of 131–132 bp were obtained using modified versions of primers described by Moreno et al [16]. Detection of the wild‐type allele used a G‐specific forward primer 5′‐CCGGCTACCCCTGCTCGTG‐3′ and the common reverse primer 5′‐CCGGCTCCCGTTCACCTG‐3′. Detection of the null allele that prevents secretion used an A‐specific forward primer 5′‐ACCGGCTACCCCTGCTCGTA‐3′ with the common reverse primer. Samples with discrepant results for genotyping and Lewis serology underwent sequencing of the *FUT2* and *FUT3* coding regions using primers described by King et al 2019 [17].

### 2.6 ABO genotyping by allelic discrimination

ABO genotype of the DISCOVER DNA samples was determined using three allelic discrimination assays to assess the polymorphisms at positions 261 (+/‐G), 526 (C/G), and 703 (G/A) of the *ABO* gene. The assay for position 526 was the TaqMan SNP genotyping assay C__27859399_10 (SNP ID rs7853989) (ThermoFisher Scientific). The other two assays used primers and TaqMan probes designed by Molecular Diagnostics, NHSBT. Sequences are available on request from the authors. All assays were run in 20μl volumes on a real‐time PCR system, according to manufacturer's instructions (Applied Biosystems).

### 2.7 Statistics

Statistical analysis were performed with the use of R (v4.0.0) [18] and respective “pubh” package (v1.3.2) [19]. Statistical comparisons for discrete variables were performed using the two‐tailed Fisher's exact test, and statistical comparisons for continuous variables were performed with the Wilcoxon rank sum test using blood group O as the baseline for comparison. Where necessary, ABO and secretor frequencies were compared using Pearson's chi‐square test against frequencies reported in the official statistics provided by NHS Blood and Transplant, comprising blood group distribution in England.

## 3 RESULTS

### 3.1 COVID‐19 patients with Blood Group A are more likely to be hospitalized and suffer cardiovascular complications

A total of 471 adult patients had been admitted to North Bristol NHS Trust (UK) with a positive PCR result for SARS‐CoV‐2, and ABO blood group data were available for 44% (*n* = 209) of these. Retrospective analysis of these data revealed that among all the blood types, blood type A was the most common in COVID‐19 patients, followed by O, B, and then type AB as the least common (**Tables** **1** and **S1**). As observed in other similar studies, the proportion of blood group A in patients with COVID‐19 was significantly higher than those with blood group O; with 105 (50.2%) patients with type A blood and 83 (39.7%) with type O (RR = 1.27, CI 95% [1.03, 1.55], *p* = 0.0246) (**Table** **2**). By comparison, in the English donor population, type O is the most common blood group accounting for 48% of the population, while type A accounts for 38% of the population [20]. The increased risk of hospitalization for blood group A for COVID‐19 is significant and is accompanied by a significantly higher instance of respiratory failure on admission, requiring ventilation (*p* = 0.01525). However, there was no significant association with length of stay in hospital or patient death rate. Hospitalized type A and O COVID‐19 patients also had a similar age range (average +/‐ SD for A = 74.5 +/‐ 16.9 and O = 72.6 +/‐ 17.9). Interestingly, while the distribution of ethnicities was near‐identical between type A and type O individuals, we observed a striking gender distribution in this study (**Tables** **1** **and** **2**) with 64% of males in the type A group compared to 43% with O type and conversely, more females with type O compared to A (56% vs. 35%) in this cohort. These observations match previous reports of male gender as a risk factor for COVID‐19 [21], but this variation is unexplained in the context of blood type.

**TABLE 1:** Retrospective analysis of critically ill patients admitted to the intensive care in North Bristol NHS Trust (UK) with a positive PCR result for SARS‐CoV‐2 and that for whom ABO blood group data were available

	**ABO Blood Group**				
	**A**	**AB**	**B**	**O**	**Total**
**Number of patients/percentage of total**	105 (50.24%)	3 (1.44%)	18 (8.61%)	83 (39.71%)	209 (100%)
**Age (±SD, years)**	74.5 ± 16.9	87.8 ± 2.8	68.3 ± 17.6	72.6 ± 17.9	73.4 ± 17.3
**Gender**					
Female	37 (35.2%)	0 (0.0%)	11 (61.1%)	47 (56.6%)	95 (45.5%)
Male	68 (64.8%)	3 (100.0%)	7 (38.9%)	36 (43.4%)	114 (54.5%)
**Ethnicity**					
Unknown/no data	5 (4.8%)	0 (0.0%)	3 (16.7%)	10 (12.0%)	18 (8.6%)
Caucasian	94 (89.5%)	3 (100.0%)	15 (83.3%)	71 (85.5%)	183 (87.6%)
Non‐Caucasian	6 (5.7%)	0 (0.0%)	0 (0.0%)	2 (2.4%)	8 (3.8%)
**Respiratory failure at admission**					
No	58 (55.2%)	3 (100.0%)	11 (61.1%)	61 (73.5%)	133 (63.6%)
Yes	47 (44.8%)	0 (0.0%)	7 (38.9%)	22 (26.5%)	76 (36.4%)
**Length of hospital stay (±SD, days)**	16.3 ± 17.6	17.0 ± 13.9	16.9 ± 25.0	16.0 ± 20.4	16.2 ± 19.3
**Inpatient death**					
Unknown/no data	2 (1.9%)	0 (0.0%)	0 (0.0%)	0 (0.0%)	2 (1.0%)
No	72 (68.6%)	2 (66.7%)	15 (83.3%)	57 (68.7%)	146 (69.9%)
Yes	31 (29.5%)	1 (33.3%)	3 (16.7%)	26 (31.3%)	61 (29.2%)
**Acute renal failure**					
No	74 (70.5%)	1 (33.3%)	12 (66.7%)	63 (75.9%)	150 (71.8%)
Yes	31 (29.5%)	2 (66.7%)	6 (33.3%)	20 (24.1%)	59 (28.2%)
**Liver dysfunction**					
No	95 (90.5%)	3 (100.0%)	16 (88.9%)	74 (89.2%)	188 (90.0%)
Yes	10 (9.5%)	0 (0.0%)	2 (11.1%)	9 (10.8%)	21 (10.0%)
**ARDS (acute respiratory distress syndrome)**					
No	94 (89.5%)	3 (100.0%)	14 (77.8%)	76 (91.6%)	187 (89.5%)
Yes	11 (10.5%)	0 (0.0%)	4 (22.2%)	7 (8.4%)	22 (10.5%)
Cardiovascular complication*					
No	75 (71.4%)	3 (100.0%)	16 (88.9%)	70 (84.3%)	164 (78.5%)
Yes	30 (28.6%)	0 (0.0%)	2 (11.1%)	13 (15.7%)	45 (21.5%)
**No complications**					
No	77 (73.3%)	2 (66.7%)	9 (50.0%)	48 (57.8%)	136 (65.1%)
Yes	28 (26.7%)	1 (33.3%)	9 (50.0%)	35 (42.2%)	73 (34.9%)

*Encompasses non‐ST‐elevation myocardial infarction (NSTEMI), ST‐elevation myocardial infarction (STEMI), atrial fibrillation, stroke, brain hemorrhage, deep vein thrombus (DVT), pulmonary embolus (PE) and congestive heart failure.

**TABLE 2:** Statistical analysis of data from Table 1

	**ABO Blood Group**			**Risk ratio (95% CI)**	***p*‐value** ^†^
	**A**	**O**	**Total**		
**Number of patients/percentage of total**	105 (55.85%)	83 (44.15%)	188 (100%)	1.27 (1.03, 1.55)	**0.0245** ^‡^
**Age (±SD, years)**	74.5 ± 16.9	72.6 ± 17.9	73.7 ± 17.3	————‐	0.4058
**Gender**					
Female	37 (35.2%)	47 (56.6%)	84 (44.7%)	1.49 (1.12, 2.00)	**0.005413**
Male	68 (64.8%)	36 (43.4%)	104 (55.3%)		
**Ethnicity**					
Unknown/no data	5 (4.8%)	10 (12.1%)	15 (8.0%)		
Caucasian	94 (89.5%)	71 (85.5%)	165 (87.8%)	0.97 (0.91, 1.02)	0.2867
Non‐Caucasian	6 (5.7%)	2 (2.4%)	8 (4.2%)		
**Respiratory failure at admission**					
No	58 (55.2%)	61 (73.5%)	119 (63.3%)	1.69 (1.11, 2.56)	**0.01525**
Yes	47 (44.8%)	22 (26.5%)	69 (36.7%)		
**Length of hospital stay (±SD, days)**	16.3 ± 17.6	16.0 ± 20.4	16.2 ± 18.8	————‐	0.3541
**Inpatient death**					
Unknown/no data	2 (1.9%)	0 (0.0%)	2 (1.1%)	0.96 (0.62, 1.49)	0.6338
No	72 (68.6%)	57 (68.7%)	129 (68.6%)		
Yes	31 (29.5%)	26 (31.3%)	57 (30.3%)		
**Acute renal failure**					
No	74 (70.5%)	63 (75.9%)	137 (72.9%)	1.22 (0.76, 2.00)	0.5054
Yes	31 (29.5%)	20 (24.1%)	51 (27.1%)		
**Liver dysfunction**					
No	95 (90.5%)	74 (89.2%)	169 (89.9%)	0.88 (0.37, 2.04)	0.9566
Yes	10 (9.5%)	9 (10.8%)	19 (10.1%)		
**ARDS (acute respiratory distress syndrome)**					
No	94 (89.5%)	76 (91.6%)	170 (90.4%)	0.81 (0.33, 1.99)	0.8235
Yes	11 (10.5%)	7 (8.4%)	18 (9.6%)		
Cardiovascular complication*					
No	75 (71.4%)	70 (84.3%)	145 (77.1%)	1.82 (1.02, 3.23)	**0.05515**
Yes	30 (28.6%)	13 (15.7%)	43 (22.9%)		
**No complications**					
No	77 (73.3%)	48 (57.8%)	125 (66.5%)	0.63 (0.42, 0.95)	**0.03749**
Yes	28 (26.7%)	35 (42.2%)	63 (33.5%)		
**Rh status**					
RhD^‐^	13 (12.4%)	16 (19.3%)	29 (15.4%)	1.09 (0.95, 1.23)	0.2728
RhD^+^	92 (87.6%)	67 (80.7%)	159 (84.6%)		

* Encompasses non‐ST‐elevation myocardial infarction (NSTEMI), ST‐elevation myocardial infarction (STEMI), atrial fibrillation, stroke, brain hemorrhage, deep vein thrombus (DVT), pulmonary embolus (PE) and congestive heart failure.

^†^ Statistical comparisons for discrete variables were performed with Fisher's Exact Test, and statistical comparisons for continuous variables were performed with the Wilcoxon rank sum test. Blood group O is used as the baseline for comparison.

^‡^ Compared against the official statistics provided by NHS Blood and Transplant, comprising blood group distribution in the United Kingdom.

Importantly, we observe a trend between blood group A status and complications with cardiovascular disease (**Tables** **1**, **2**, and S1). Individuals with blood group type A displayed almost double the risk of suffering from a cardiovascular complication compared to individuals with type O (RR = 1.82, CI 95% [1.02, 3.23], *p* = 0.055), and the main contributor to this effect is congestive heart failure (Risk Ratio (RR) = 2.24, Confidence Interval (CI) 95% [0.92, 5.43], *p* = 0.074). In contrast, there was no observed association between A and O blood group and suffering from acute respiratory distress syndrome (ARDS) (RR = 0.81, CI 95% [0.33,1.99], *p* = 0.8235). As a control comparison, we did not detect a significant difference in the frequency of RhD phenotype between A and O (RR = 1.09, CI 95% [0.95, 1.23], *p* = 0.273). Further statistical analysis was performed by dividing A and O type populations into two subgroups by gender (**Table** **3**). While the risk ratios remained supportive of the hypothesis that the A blood group is associated with cardiovascular complications in COVID‐19 patients regardless of gender (Male: RR = 1.59, CI 95% [0.75,3.33], *p* = 0.3083; Female: RR = 1.92, CI 95% [0.75, 4.76], *p* = 0.2774), the two comparisons were not statistically meaningful, likely due to the decrease in number of patients per compared group.

**TABLE 3:** Statistical analysis of patients with cardiovascular complications, split into male and female, from table 1

	**ABO blood group**			
	**A**	**O**	**Total**	**Risk ratio (95% CI)**
Cardiovascular complication*, male				
No	47 (69.1%)	29 (80.6%)	76 (73.1%)	1.59 (0.75, 3.33)
Yes	21 (30.9%)	7 (19.4%)	28 (26.9%)	
Cardiovascular complication*, female				
No	28 (75.7%)	41 (87.2%)	69 (82.1%)	1.92 (0.75, 4.76)
Yes	9 (24.3%)	6 (12.8%)	15 (17.9%)	

*Cardiovascular complications encompasses non‐ST‐elevation myocardial infarction (NSTEMI), ST‐elevation myocardial infarction (STEMI), new episode atrial fibrillation, stroke or brain hemorrhage, deep vein thrombus (DVT), pulmonary embolus (PE) and new/worsening congestive heart failure.

†Statistical comparisons for discrete variables were performed with Fisher's Exact Test, and statistical comparisons for continuous variables were performed with the Wilcoxon rank sum test. Blood group O is used as the baseline for comparison.

### 3.2 Secretor status is a compounding risk factor for hospitalization of group A COVID‐19 patients

To further investigate the association of COVID‐19 with blood group type, blood samples collected by the DISCOVER study were tested for ABO group and secretor status using DNA‐based methodologies (**Table** **4**. **Table S2** for clinical data on admission). While the two cohorts have different inclusion criteria, a retrospective analysis showed 24 patients were included in both cohorts, and these comprised approximately 10% of the Avon CAP cohort and 20% of the DISCOVER cohort. We observed a similar trend for the association of blood group A with cardiovascular complications in COVID‐19 as compared to O which is significant (RR = 2.00, CI 95% [1.01, 3.95], *p* = 0.074). When the laboratory blood group data from non‐overlapping patients confirmed PCR positive for SARS‐CoV2 were combined with the earlier health surveillance data, (*n* = 275) the association of blood group A with hospitalization (RR = 1.24, CI 95% [1.05, 1.47], *p* = 0.0111) and blood group A with cardiovascular complications (RR = 2.56, CI 95% [1.43, 4.55], *p* = 0.0011) now has increased significance. (**Tables S3** **and S** **4**). The blood group A was associated with cardiovascular complications in COVID‐19 patients regardless of gender and was significant with the larger group of patients (Male: RR = 2.50, CI 95% [1.18, 5.26], *p* = 0.0168; Female: RR = 2.33, CI 95% [0.95, 5.88], *p *= 0.0919).

**TABLE 4:** Retrospective analysis of patients admitted to North Bristol NHS Trust (UK) and enrolled onto the DISCOVER study, that were phenotyped and genotyped for ABO blood group and for secretor status. Highlighted in bold are cardiovascular complications as a result of COVID19 infection. Cardiovascular complications are classed as patients requiring or developing inotropic support, NSTEMI, STEMI, myocarditis, new episode of atrial fibrillation, new or worsening congestive heart failure or new DVT/PE

	**ABO Blood Groups**					**Secretor phenotype**		
	A	AB	B	O	Total	Non‐secretor	Secretor	Total
	(*N* = 62)	(*N* = 8)	(*N* = 20)	(*N* = 53)	(*N* = 143)	(*N* = 26)	(*N* = 117)	(*N* = 143)
**ABO**								
A						5 (19.2%)	57 (48.7%)	62 (43.4%)
AB						3 (11.5%)	5 (4.3%)	8 (5.6%)
B						5 (19.2%)	15 (12.8%)	20 (14.0%)
O						13 (50.0%)	40 (34.2%)	53 (37.1%)
**PCR Secretor**								
Non‐secretor	5 (8.1%)	3 (37.5%)	5 (25.0%)	13 (24.5%)	26 (18.2%)			
Secretor	57 (91.9%)	5 (62.5%)	15 (75.0%)	40 (75.5%)	117 (81.8%)			
**Age**	60.5 ± 18.3	63.6 ± 17.4	52.8 ± 11.4	58.1 ± 14.5	58.7 ± 16.1	58.0 ± 12.7	58.9 ± 16.8	58.7 ± 16.1
**Sex**								
Female	27 (43.5%)	2 (25.0%)	8 (40.0%)	21 (39.6%)	58 (40.6%)	13 (50.0%)	45 (38.5%)	58 (40.6%)
Male	35 (56.5%)	6 (75.0%)	12 (60.0%)	32 (60.4%)	85 (59.4%)	13 (50.0%)	72 (61.5%)	85 (59.4%)
**Caucasian**								
No	9 (14.5%)	1 (12.5%)	5 (25.0%)	6 (11.3%)	21 (14.7%)	5 (19.2%)	16 (13.7%)	21 (14.7%)
Unknown	6 (9.7%)	3 (37.5%)	3 (15.0%)	5 (9.4%)	17 (11.9%)	6 (23.1%)	11 (9.4%)	17 (11.9%)
Yes	47 (75.8%)	4 (50.0%)	12 (60.0%)	42 (79.2%)	105 (73.4%)	15 (57.7%)	90 (76.9%)	105 (73.4%)
**Preconditions diabetes**								
No	47 (75.8%)	7 (87.5%)	16 (80.0%)	43 (81.1%)	113 (79.0%)	22 (84.6%)	91 (77.8%)	113 (79.0%)
T1DM	2 (3.2%)	0 (0.0%)	0 (0.0%)	1 (1.9%)	3 (2.1%)	0 (0.0%)	3 (2.6%)	3 (2.1%)
T2DM	13 (21.0%)	1 (12.5%)	4 (20.0%)	9 (17.0%)	27 (18.9%)	4 (15.4%)	23 (19.7%)	27 (18.9%)
**Preconditions heart disease**								
No	42 (67.7%)	6 (75.0%)	17 (85.0%)	44 (83.0%)	109 (76.2%)	20 (76.9%)	89 (76.1%)	109 (76.2%)
Yes	20 (32.3%)	2 (25.0%)	3 (15.0%)	9 (17.0%)	34 (23.8%)	6 (23.1%)	28 (23.9%)	34 (23.8%)
**Preconditions hypertension**								
No	41 (66.1%)	7 (87.5%)	15 (75.0%)	38 (71.7%)	101 (70.6%)	19 (73.1%)	82 (70.1%)	101 (70.6%)
Yes	21 (33.9%)	1 (12.5%)	5 (25.0%)	15 (28.3%)	42 (29.4%)	7 (26.9%)	35 (29.9%)	42 (29.4%)
**Preconditions chronic lung disease**								
No	43 (69.4%)	6 (75.0%)	12 (60.0%)	44 (83.0%)	105 (73.4%)	18 (69.2%)	87 (74.4%)	105 (73.4%)
Yes	19 (30.6%)	2 (25.0%)	8 (40.0%)	9 (17.0%)	38 (26.6%)	8 (30.8%)	30 (25.6%)	38 (26.6%)
**Preconditions severe liver disease**								
No	61 (98.4%)	8 (100.0%)	20 (100.0%)	52 (98.1%)	141 (98.6%)	26 (100.0%)	115 (98.3%)	141 (98.6%)
Yes	1 (1.6%)	0 (0.0%)	0 (0.0%)	1 (1.9%)	2 (1.4%)	0 (0.0%)	2 (1.7%)	2 (1.4%)
**Preconditions severe kidney impairment**								
No	58 (93.5%)	7 (87.5%)	20 (100.0%)	44 (83.0%)	129 (90.2%)	25 (96.2%)	104 (88.9%)	129 (90.2%)
Yes	4 (6.5%)	1 (12.5%)	0 (0.0%)	9 (17.0%)	14 (9.8%)	1 (3.8%)	13 (11.1%)	14 (9.8%)
**Symptoms respiratory**								
	11 (17.7%)	0 (0.0%)	0 (0.0%)	6 (11.3%)	17 (11.9%)	0 (0.0%)	17 (14.5%)	17 (11.9%)
Yes	51 (82.3%)	8 (100.0%)	20 (100.0%)	47 (88.7%)	126 (88.1%)	26 (100.0%)	100 (85.5%)	126 (88.1%)
**Symptoms systemic**								
No	20 (32.3%)	1 (12.5%)	1 (5.0%)	5 (9.4%)	27 (18.9%)	3 (11.5%)	24 (20.5%)	27 (18.9%)
Yes	42 (67.7%)	7 (87.5%)	19 (95.0%)	48 (90.6%)	116 (81.1%)	23 (88.5%)	93 (79.5%)	116 (81.1%)
**Symptoms neurological**								
No	54 (87.1%)	6 (75.0%)	18 (90.0%)	47 (88.7%)	125 (87.4%)	22 (84.6%)	103 (88.0%)	125 (87.4%)
Yes	8 (12.9%)	2 (25.0%)	2 (10.0%)	6 (11.3%)	18 (12.6%)	4 (15.4%)	14 (12.0%)	18 (12.6%)
**General outcome**								
Deceased	5 (8.1%)	1 (12.5%)	2 (10.0%)	3 (5.7%)	11 (7.7%)	2 (7.7%)	9 (7.7%)	11 (7.7%)
Discharged	52 (83.9%)	7 (87.5%)	15 (75.0%)	45 (84.9%)	119 (83.2%)	21 (80.8%)	98 (83.8%)	119 (83.2%)
Inpatient	3 (4.8%)	0 (0.0%)	0 (0.0%)	2 (3.8%)	5 (3.5%)	0 (0.0%)	5 (4.3%)	5 (3.5%)
Unknown	2 (3.2%)	0 (0.0%)	3 (15.0%)	3 (5.7%)	8 (5.6%)	3 (11.5%)	5 (4.3%)	8 (5.6%)
**Complications cardiovascular**								
No	49 (79.0%)	8 (100.0%)	19 (95.0%)	52 (98.1%)	128 (89.5%)	223 (92.3%)	104 (88.9%)	128 (89.5%)
**Yes**	**13 (21.0%)**	**0 (0.0%)**	**1 (5.0%)**	**1 (1.9%)**	**15 (10.5%)**	**3 (7.7%)**	**13 (11.1%)**	**15 (10.5%)**
**Complications acute renal failure**								
No	57 (91.9%)	8 (100.0%)	18 (90.0%)	46 (86.8%)	129 (90.2%)	23 (88.5%)	106 (90.6%)	129 (90.2%)
Yes	5 (8.1%)	0 (0.0%)	2 (10.0%)	7 (13.2%)	14 (9.8%)	3 (11.5%)	11 (9.4%)	14 (9.8%)
**Complications liver dysfunction**								
No	50 (80.6%)	7 (87.5%)	19 (95.0%)	46 (86.8%)	122 (85.3%)	22 (84.6%)	100 (85.5%)	122 (85.3%)
Yes	12 (19.4%)	1 (12.5%)	1 (5.0%)	7 (13.2%)	21 (14.7%)	4 (15.4%)	17 (14.5%)	21 (14.7%)

In epithelial tissues and secretions, ABO expression is heavily dependent on the inheritance of the Secretor *Se/FUT2* gene which can also be protective against viral infection. Due to mutations in other fucosyltransferase genes, individuals can also be Le^a−b−^ and rarely Le^a+b+^ so secretor status cannot always be determined by red cell typing alone. We therefore conducted DNA analysis on this cohort to further determine ABO and secretor status and subsequently compared all symptom and laboratory values to ABO and secretor genotype. Individuals can either be secretors (SeSe or Sese) or non‐secretors (sese). Strikingly, we observed that the vast majority of blood group A patients expressed an active Secretor gene (*FUT2*), with the incidence of non‐secretors being significantly lower than would be expected from comparison with the normal distribution in the general population (**Table** **4**, 8.1% vs. 20%, *p* = 0.019) [22]. No initial correlation with Se genotype (SeSe versus Sese) and disease outcome was observed. After additional blood group genotype analysis, this showed there were three deaths among only nine AA Se/Se or Se/se patients compared with two deaths from 53 AO Se/Se or Se/se patients (**Table** **5**), but we caution any extrapolation from this result as the sample size is small.

**TABLE 5:** Retrospective analysis of patients admitted to North Bristol NHS Trust (UK) and enrolled onto the DISCOVER study, that were genotyped for ABO blood group and secretor status. Highlighted in bold are cardiovascular complications as a result of COVID19 infection. Cardiovascular complications are classed as patients requiring or developing inotropic support, NSTEMI, STEMI, myocarditis, new episode of atrial fibrillation, new or worsening congestive heart failure or new DVT/PE

	**ABO Genotype**							**Secretor genotype**			
	AA	AO	AB	BB	BO	OO	Total	sese	SeSe	Sese	Total
	(*N* = 9)	(*N* = 53)	(*N* = 8)	(*N* = 1)	(*N* = 18)	(*N* = 53)	(*N* = 142)	(*N* = 25)	(*N* = 47)	(*N* = 70)	(*N* = 142)
**ABO**											
AA								1 (4.0%)	0 (0.0%)	8 (11.4%)	9 (6.3%)
AO								4 (16.0%)	18 (38.3%)	31 (44.3%)	53 (37.3%)
AB								3 (12.0%)	0 (0.0%)	5 (7.1%)	8 (5.6%)
BB								0 (0.0%)	0 (0.0%)	1 (1.4%)	1 (0.7%)
BO								4 (16.0%)	9 (19.1%)	5 (7.1%)	18 (12.7%)
OO								13 (52.0%)	20 (42.6%)	20 (28.6%)	53 (37.3%)
**PCR Secretor**											
sese (non‐secretor)	1 (11.2%)	4 (7.5%)	3 (37.5%)	0 (0.0%)	4 (22.2%)	13 (24.5%)	25 (17.6%)				
Sese (secretor)	8 (88.8%)	31 (58.5%)	5 (62.5%)	1 (100%)	5 (27.8%)	20 (37.7%)	70 (49.3%)				
SeSe (secretor)	0 (0.0%)	18 (34.0%)	0 (0.0%)	0 (0.0%)	9 (50.0%)	20 (37.7%)	47 (33.1%)				
**Age**	62.9 ± 26.9	59.9 ± 39.9	63.6 ± 17.4	31 ± 0	54.4 ± 12.4	58.1 ± 14.5	58.7 ± 16.1	58.1 ± 12.7	61.6 ± 31.3	57.1 ± 30.9	58.7 ± 16.1
**Sex**											
Female	2 (22.2%)	25 (47.2%)	2 (25.0%)	0 (0.0%)	7 (38.9%)	21 (39.6%)	57 (40.1%)	12 (48.0%)	20 (42.6%)	25 (35.7%)	57 (40.1%)
Male	7 (77.8%)	28 (52.8%)	6 (75.0%)	1 (100%)	11 (61.1%)	32 (60.4%)	85 (59.9%)	13 (52.0%)	27 (57.4%)	45 (64.3%)	85 (59.9%)
**Caucasian**											
No	0 (0.0%)	9 (17.0%)	1 (12.5%)	0 (0.0%)	4 (22.2%)	42 (79.2%)	56 (39.4%)	5 (20.0%)	7 (14.9%)	8 (11.4%)	20 (14.1%)
Unknown	2 (22.2%)	4 (7.5%)	3 (37.5%)	0 (0.0%)	3 (16.7%)	5 (9.4%)	17 (12.0%)	5 (20.0%)	6 (12.8%)	6 (8.6%)	17 (12.0%)
Yes	7 (77.8%)	40 (75.5%)	4 (50.0%)	1 (100%)	11 (61.1%)	6 (11.3%)	69 (48.6%)	15 (60.0%)	34 (72.3%)	56 (80.0%)	105 (73.9%)
**Preconditions diabetes**											
No	8 (88.8%)	39 (73.6%)	7 (87.5%)	1 (100%)	14 (77.8%)	43 (81.1%)	112 (78.9%)	21 (84.0%)	33 (70.2%)	58 (82.9%)	112 (78.9%)
T1DM	0 (0.0%)	2 (3.8%)	0 (0.0%)	0 (0.0%)	0 (0.0%)	1 (1.9%)	3 (2.1%)	0 (0.0%)	1 (2.1%)	2 (2.9%)	3 (2.1%)
T2DM	1 (11.2%)	12 (22.6%)	1 (12.5%)	0 (0.0%)	4 (22.2%)	9 (17.0%)	27 (19.0%)	4 (16.0%)	13 (27.7%)	10 (14.3%)	27 (19.0%)
**Preconditions heart disease**											
No	5 (55.5%)	37 (70.0%)	6 (75.0%)	1 (100%)	15 (83.3%)	44 (83.0%)	108 (76.1%)	20 (80.0%)	38 (80.9%)	51 (71.9%)	109 (76.8%)
Yes	4 (44.5%)	16 (30%)	2 (25.0%)	0 (0.0%)	3 (16.7%)	9 (17.0%)	34 (23.9%)	5 (20.0%)	9 (19.1)	19 (27.1%)	33 (23.2%)
**Preconditions hypertension**											
No	5 (55.5%)	36 (67.9%)	7 (87.5%)	1 (100%)	13 (72.2%)	38 (71.7%)	100 (70.4%)	18 (72.0%)	32 (68.1%)	50 (71.4%)	100 (70.4%)
Yes	4 (44.5%)	17 (32.1%)	1 (12.5%)	0 (0.0%)	5 (27.8%)	15 (28.3%)	42 (29.6%)	7 (28.0%)	15 (31.9%)	20 (28.6%)	42 (29.6%)
**Preconditions chronic lung disease**											
No	7 (77.8%)	36 (67.9%)	6 (75.0%)	1 (100%)	10 (55.6%)	44 (83.0%)	104 (73.2%)	17 (68.0%)	33 (70.2%)	54 (77.1%)	104 (73.2%)
Yes	2 (22.2%)	17 (32.1%)	2 (25.0%)	0 (0.0%)	8 (44.4%)	9 (17.0%)	38 (26.8%)	8 (32.0%)	14 (29.8%)	16 (22.9%)	38 (26.8%)
**Preconditions severe liver disease**											
No	9 (100%)	52 (98.2%)	8 (100%)	1 (100%)	18 (100%)	52 (98.1%)	140 (98.6%)	25 (100%)	47 (100%)	68 (97.1%)	140 (98.6%)
Yes	0 (0.0%)	1 (1.8%)	0 (0.0%)	0 (0.0%)	0 (0.0%)	1 (1.9%)	2 (1.4%)	0 (0.0%)	0 (0.0%)	2 (2.9%)	2 (1.4%)
**Preconditions severe kidney impairment**											
No	8 (88.8%)	50 (94.3%)	7 (87.5%)	1 (100%)	18 (100%)	44 (83.0%)	128 (90.1%)	24 (96.0%)	41 (87.2%)	63 (90.0%)	128 (90.1%)
Yes	1 (11.2%)	3 (5.7%)	1 (12.5%)	0 (0.0%)	0 (0.0%)	9 (17.0%)	14 (9.9%)	1 (4.0%)	6 (12.8%)	7 (10.0%)	14 (9.9%)
**Symptoms respiratory**											
No	2 (22.2%)	9 (17.0%)	0 (0.0%)	0 (0.0%)	18 (100%)	6 (11.3%)	35 (24.6%)	0 (0.0%)	5 (10.6%)	12 (17.1%)	17 (12.0%)
Yes	7 (77.7%)	44 (83.0%)	8 (100%)	1 (100%)	0 (0.0%)	47 (88.7%)	107 (75.4%)	25 (100%)	42 (89.4%)	58 (82.9%)	125 (88.0%)
**Symptoms systemic**											
No	2 (22.2%)	18 (34.0%)	1 (12.5%)	0 (0.0%)	1 (5.6%)	5 (9.4%)	27 (19%)	2 (8.0%)	10 (21.3%)	14 (20.0%)	26 (18.3%)
Yes	7 (77.8%)	35 (66.0%)	7 (87.5%)	1 (100%)	17 (94.4%)	48 (90.6%)	115 (81%)	23 (92.0%)	37 (78.7%)	56 (80.0%)	116 (81.7%)
**Symptoms neurological**											
No	8 (88.8%)	46 (86.8%)	6 (75.0%)	1 (100%)	16 (88.9%)	47 (88.7%)	124 (87.3%)	21 (84.0%)	40 (85.1%)	63 (90.0%)	124 (87.4%)
Yes	1 (11.2%)	7 (13.2%)	2 (25.0%)	0 (0.0%)	2 (11.1%)	6 (11.3%)	18 (12.7%)	4 (16.0%)	7 (14.9%)	7 (10.0%)	18 (12.7%)
**General outcome**											
Deceased	3 (33.3%)	2 (3.8%)	0 (0.0%)	0 (0.0%)	2 (11.1%)	3 (5.7%)	10 (7.0%)	1 (4.0%)	2 (4.3%)	7 (10.0%)	10 (7.0%)
Discharged	5 (55.5%)	47 (88.7%)	7 (87.5%)	1 (100%)	13 (72.2%)	45 (84.9%)	118 (83.1%)	21 (84.0%)	40 (85.1%)	57 (81.4%)	118 (83.1%)
Inpatient	0 (0.0%)	3 (5.6%)	0 (0.0%)	0 (0.0%)	0 (0.0%)	2 (3.8%)	5 (3.5%)	0 (0.0%)	0 (0.0%)	4 (5.7%)	4 (2.8%)
Unknown	1 (11.2%)	1 (1.8%)	1 (12.5%)	0 (0.0%)	3 (16.7%)	3 (5.7%)	9 (6.3%)	3 (12.0%)	5 (10.6%)	2 (2.9%)	10 (7.0%)
**Complications cardiovascular**											
No	7 (77.8%)	42 (77.3%)	8 (100%)	1 (100%)	17 (94.4%)	52 (98.1%)	127 (89.4%)	22 (88.0%)	42 (89.4%)	62 (88.6%)	126 (88.7%)
Yes	**2 (22.2%)**	**11 (20.7%)**	**0 (0.0%)**	**0 (0.0%)**	**1 (5.6%)**	**1 (1.9%)**	**15 (10.6%)**	**3 (12.0%)**	**5 (10.6%)**	**8 (11.4%)**	**16 (11.3%)**
**Complications acute renal failure**											
No	8 (88.8%)	49 (92.4%)	8 (100%)	1 (100%)	16 (88.9%)	46 (86.8%)	128 (90.1%)	22 (88.0%)	41 (87.2%)	65 (92.9%)	128 (90.1%)
Yes	1 (11.2%)	4 (7.5%)	0 (0.0%)	0 (0.0%)	2 (11.1%)	7 (13.2%)	14 (9.9%)	3 (12.0%)	6 (12.8%)	5 (7.1%)	14 (9.9%)
**Complications liver dysfunction**											
No	8 (88.8%)	42 (79.2%)	7 (87.5%)	1 (100%)	17 (94.4%)	46 (86.8%)	121 (86.2%)	21 (84.0%)	41 (87.2%)	59 (84.3%)	121 (85.2%)
Yes	1 (11.2%)	11(20.8%)	1 (12.5%)	0 (0.0%)	1 (5.6%)	7 (13.2%)	21 (14.8%)	4 (16.0%)	6 (12.8%)	11 (15.7%)	21 (14.8%)

In order to determine whether non‐secretor status among group A individuals was associated with increased protection against SARS‐CoV‐2 or whether it simply reduced disease severity, residual testing samples were accessed from the UK wide NHSBT convalescent plasma donations collected from recovered COVID‐19 patients [23]. The convalescent plasma samples include a broader range of donors who had self‐reported recovery from hospitalization, donors who had positive PCR tests and donors who had suffered known symptoms and undergone a positive antibody test. In confirmation of our previous results, we observed a lower than expected number of non‐secretors in blood group A donors who had reported hospitalization (*N* = 55), but note that this analysis is based on a small sample size (**Table** **6**). Importantly, across all convalescent donors sampled, we observed the anticipated number of non‐secretors. However, in patients with blood type A, a higher‐than‐expected number of non‐secretors was identified (25% vs. 20%, *p* = 0.01) (**Table** **6**). Taken together, the DISCOVER study and convalescent plasma donor study results suggest that blood type A non‐secretors are not necessarily protected from SARS‐CoV‐2 infection but may experience less severe disease.

**TABLE 6:** ABO and secretor phenotype of patients admitted to North Bristol NHS Trust (UK) and enrolled onto the DISCOVER study, samples from known hospitalized COVID19 NHSBT convalescent plasma donations and a much large cohort of non‐hospitalized NHSBT convalescent plasma donations. Highlighted in bold are the numbers and percentages of secretors and non‐secretors of blood type A in each cohort

	**DISCOVER**				
	A	AB	B	O	Total
	(*N* = 62)	(*N* = 8)	(*N* = 20)	(*N* = 53)	(*N* = 143)
**PCR secretor**					
Non‐secretor	**5 (8.1%)**	3 (37.5%)	5 (25.0%)	13 (24.5%)	26 (18.2%)
Secretor	**57 (91.9%)**	5 (62.5%)	15 (75.0%)	40 (75.5%)	117 (81.8%)

	**Hospitalized convalescent plasma**				
	A	AB	B	O	Total
	(*N* = 23)	(*N* = 4)	(*N* = 6)	(*N* = 22)	(*N* = 55)
**PCR secretor**					
Non‐secretor	**3 (13.0%)**	1 (25.0%)	2 (33.3%)	5 (22.7%)	11 (20.0%)
Secretor	**20 (87.0%)**	3 (75.0%)	4 (66.6%)	17 (77.3%)	44 (80.0%)

	**Non‐hospitalized convalescent plasma**				
	A	AB	B	O	Total
	(*N* = 392)	*(N* = 42)	(*N* = 97)	(*N* = 397)	(*N* = 928)
**PCR secretor**					
Non‐secretor	**101 (25.8%)**	8 (19.0%)	19 (19.6%)	93 (23.4%)	221 (23.8%)
Secretor	**291 (74.2%)**	34 (81.0%)	78 (80.4%)	304 (76.6%)	707 (76.2%)

## 4 DISCUSSION

Studies carried out in hospitalized SARS‐CoV‐2 patients in China were the first to link blood group A with greater susceptibility to COVID‐19 compared to blood group O [15]. Since then, multiple other studies carried out in other countries have supported an association between ABO type and SARS‐CoV‐2 susceptibility to infection and/or outcomes [24, 25, 26, 27]. The reasons for the association of severe COVID‐19 with blood type A are unknown, but it has been suggested that this could be caused by O group patients having anti‐A type antibodies [28], that A type glycans could function as co‐receptors for SARS‐CoV‐2 [29] or due to the known effects of blood groups on thrombosis risk due to von Willebrand factor (VWF) levels [30]. However, recently, the conclusion that ABO group influences COVID‐19 severity has been questioned [31, 32].

Here, we report a significant increased risk of hospitalization for blood group A COVID19 patients compared to patients with blood group O. This was accompanied by a significantly higher instance of respiratory failure on admission, requiring ventilation, but no significant increase in patient death. Our data link blood group A preponderance in COVID‐19 with cardiovascular outcomes. We found no association with blood group A and development of ARDS, suggesting that SARS‐CoV‐2 does not bind preferentially to the A blood group structures, as is the case in several other infectious diseases [8]. This result is consistent with studies of younger healthier populations where no bias to blood group A over group O was observed [32]. We propose that the apparently conflicting results between studies to date can be explained by the nature of the patient population studied, because many studies focus on patient populations ill enough to need hospital admission. Such individuals are mostly elderly and more likely to have comorbidities.

The association of blood group A with cardiac disease is well documented and is linked to VWF (reviewed by Ward et al [33]). Our data are the first to specifically link the severity of disease in COVID‐19 patients to homozygous group A individuals who are also secretors. ABO antigens are present on VWF, and group O VWF has been shown to have a shorter half‐life in plasma than non‐group O VWF [34] and corresponds with the observation that levels of VWF in plasma are known to be highest in homozygous group A individuals and homozygous Se individuals [35]. VWF forms high molecular multimers with increasing levels of multimerization leading to increased VWF adhesion to collagen and VWF induced platelet aggregation [36]. Plasma levels of VWF multimers are regulated by proteolytic cleavage by ADAMTS13 [37] with N‐glycosylation of VWF, particularly at position 1574 near the site of cleavage by ADAMTS13, providing some protection against proteolysis [38]. It follows that more extensive glycosylation of VWF found in group A secretors but lacking in group O secretors would also result in higher circulating levels of high molecular weight VWF multimers in Group A. In addition, it has been observed that ABO genotype is a major determinant of ABO expression on platelets with homozygous A giving the highest expression [39]; ABO expression on platelets determines how they adhere to VWF captured by exposed collagen in the endothelium [40], with non‐group O platelets adhering more strongly than group O. This leads to increased and more stable thrombus production in non‐group O individuals. Therefore, group A and homozygous Se individuals have higher plasma levels of VWF, less proteolytic cleavage of high molecular weight multimeric VWF, and group A platelets adhere more strongly to VWF. The cumulative effect would predispose homozygous Se Group A patients to cardiac problems and thrombosis during infections with SARS‐CoV‐2. The mechanism whereby infection with SARS‐CoV‐2 influences this predisposition is currently unknown, but COVID‐19 has been reported to be associated with inducing a hypercoagulable state [41]. It is known that VWF protein levels associated with pulmonary vascular endothelial cells are linked to ABO determinants [42]. We speculate that binding of SARS‐CoV‐2 virus to its receptor ACE2 in the lungs may activate endothelial cells resulting in enhanced secretion of VWF into peripheral circulation.

Although we cannot exclude the possibility that ABO and secretor status may be influencing COVID19 pathogenesis through different mechanisms, these results provide an explanation for discrepant observations reported regarding the significance of blood group A and COVID‐19 disease severity. They also indicate that determination of the genotype and secretor status of group A individuals with SARS‐CoV‐2 infection could be a useful diagnostic aid to the stratification of risk of severity in hospitalized patients. More extensive studies are needed to further explore the stratification of patients by blood group and/or VWF glycosylation and thereby facilitate identification of the most at risk and to understand the complex disease mechanism that induces the hypercoagulative state.

## AUTHOR CONTRIBUTIONS

TJ Mankelow processed samples and performed serology and Se genotyping, analysed data, and wrote the paper. BK Singleton processed samples and performed serology, Se genotyping and ABO genotyping. PL Moura collated data and performed statistical analysis and wrote the paper. CJ Stevens‐Hernandez, NM Cogan, G Gyorffy, and S Kupzig processed samples and performed serology and Se genotyping. L Nichols, C Asby, J Pooley, F Moghaddas, G Ruffino, F Hosseini, C Hyams, A Noel, and A Cooper conducted health data surveillance. A Finn, D Arnold, C Hyams, and F Hamilton initiated the patient studies at North Bristol NHS trust, provided clinical information of samples, and wrote the paper. D Anstee, C Hyams, and AM Toye instigated the research and wrote the paper.

## CONFLICT OF INTEREST

The authors declare no competing financial interests.

## Supporting information

Supporting Information

## Data Availability

The data that support the findings of this study are available on request from the corresponding author. The data are not publicly available due to privacy or ethical restrictions.
