# Supplementary material for: Blood group type A secretors are associated with a higher risk of COVID‐19 cardiovascular disease complications
Source: EJHaem. 2021 Apr 2;2(2):175–87. doi: 10.1002/jha2.180 (PMC8176350; doi:10.1002/jha2.180)
Supplement: Supplementary file 1 — Supporting Information [file JHA2-2-175-s001.pdf]

	ABO blood group				
	**A**	**AB**	**B**	**O**	**Total**
**Number / Percentage of total**	105 (50.24%)	3 (1.44%)	18 (8.61%)	83 (39.71%)	209 (100%)
**Age (± SD, years)**	74.5 ± 16.9	87.8 ± 2.8	68.3 ± 17.6	72.6 ± 17.9	73.4 ± 17.3
**Gender**					
Female	37 (35.2%)	0 (0.0%)	11 (61.1%)	47 (56.6%)	95 (45.5%)
Male	68 (64.8%)	3 (100.0%)	7 (38.9%)	36 (43.4%)	114 (54.5%)
**Ethnicity**					
Unknown/No data	5 (4.8%)	0 (0.0%)	3 (16.7%)	10 (12.0%)	18 (8.6%)
Caucasian	94 (89.5%)	3 (100.0%)	15 (83.3%)	71 (85.5%)	183 (87.6%)
Non-caucasian	6 (5.7%)	0 (0.0%)	0 (0.0%)	2 (2.4%)	8 (3.8%)
**Highest Level of Care Required**					
Unknown/No data	1 (1.0%)	0 (0.0%)	0 (0.0%)	0 (0.0%)	1 (0.5%)
CCU or high-care area	9 (8.6%)	0 (0.0%)	0 (0.0%)	4 (4.8%)	13 (6.2%)
General Medical Ward	91 (86.7%)	3 (100.0%)	15 (83.3%)	75 (90.4%)	184 (88.0%)
Intensive Care/HDU	4 (3.8%)	0 (0.0%)	3 (16.7%)	4 (4.8%)	11 (5.3%)
**Ventilatory Support Required**					
CPAP	10 (9.5%)	0 (0.0%)	1 (5.6%)	4 (4.8%)	15 (7.2%)
High-Flow Nasal Cannulae	2 (1.9%)	0 (0.0%)	1 (5.6%)	2 (2.4%)	5 (2.4%)
Intubation	4 (3.8%)	0 (0.0%)	3 (16.7%)	2 (2.4%)	9 (4.3%)
None	89 (84.8%)	3 (100.0%)	13 (72.2%)	75 (90.4%)	180 (86.1%)
**Inotropic Support Required**					
Not applicable	101 (96.2%)	3 (100.0%)	15 (83.3%)	79 (95.2%)	198 (94.7%)
No	0 (0.0%)	0 (0.0%)	1 (5.6%)	1 (1.2%)	2 (1.0%)
Yes	4 (3.8%)	0 (0.0%)	2 (11.1%)	3 (3.6%)	9 (4.3%)
**Respiratory failure at admission**					
No	58 (55.2%)	3 (100.0%)	11 (61.1%)	61 (73.5%)	133 (63.6%)
Yes	47 (44.8%)	0 (0.0%)	7 (38.9%)	22 (26.5%)	76 (36.4%)
**Length of hospital stay (± SD, days)**	16.3 ± 17.6	17.0 ± 13.9	16.9 ± 25.0	16.0 ± 20.4	16.2 ± 19.3
**Inpatient death**					
Unknown/No data	2 (1.9%)	0 (0.0%)	0 (0.0%)	0 (0.0%)	2 (1.0%)
No	72 (68.6%)	2 (66.7%)	15 (83.3%)	57 (68.7%)	146 (69.9%)
Yes	31 (29.5%)	1 (33.3%)	3 (16.7%)	26 (31.3%)	61 (29.2%)
**Acute renal failure**					
No	74 (70.5%)	1 (33.3%)	12 (66.7%)	63 (75.9%)	150 (71.8%)
Yes	31 (29.5%)	2 (66.7%)	6 (33.3%)	20 (24.1%)	59 (28.2%)
**Liver dysfunction**					
No	95 (90.5%)	3 (100.0%)	16 (88.9%)	74 (89.2%)	188 (90.0%)
Yes	10 (9.5%)	0 (0.0%)	2 (11.1%)	9 (10.8%)	21 (10.0%)
**Hospital acquired infection**					
No	98 (93.3%)	2 (66.7%)	17 (94.4%)	80 (96.4%)	197 (94.3%)
Yes	7 (6.7%)	1 (33.3%)	1 (5.6%)	3 (3.6%)	12 (5.7%)
**ARDS (Acute respiratory distress syndrome)**					
No	94 (89.5%)	3 (100.0%)	14 (77.8%)	76 (91.6%)	187 (89.5%)
Yes	11 (10.5%)	0 (0.0%)	4 (22.2%)	7 (8.4%)	22 (10.5%)
**Non-ST-elevation myocardial infarction**					
No	104 (99.0%)	3 (100.0%)	18 (100.0%)	83 (100.0%)	208 (99.5%)
Yes	1 (1.0%)	0 (0.0%)	0 (0.0%)	0 (0.0%)	1 (0.5%)
**ST-elevation myocardial infarction (STEMI)**					
No	105 (100.0%)	3 (100.0%)	18 (100.0%)	83 (100.0%)	209 (100.0%)
Yes	0 (0.0%)	0 (0.0%)	0 (0.0%)	0 (0.0%)	0 (0.0%)
**Atrial fibrillation**					
No	98 (93.3%)	3 (100.0%)	18 (100.0%)	79 (95.2%)	198 (94.7%)
Yes	7 (6.7%)	0 (0.0%)	0 (0.0%)	4 (4.8%)	11 (5.3%)
**Stroke or brain haemorrhage**					
No	102 (97.1%)	3 (100.0%)	18 (100.0%)	83 (100.0%)	206 (98.6%)
Yes	3 (2.9%)	0 (0.0%)	0 (0.0%)	0 (0.0%)	3 (1.4%)
**Deep vein thrombus (DVT)**					
No	104 (99.0%)	3 (100.0%)	18 (100.0%)	80 (96.4%)	205 (98.1%)
Yes	1 (1.0%)	0 (0.0%)	0 (0.0%)	3 (3.6%)	4 (1.9%)
**Pulmonary embolus (PE)**					
No	103 (98.1%)	3 (100.0%)	17 (94.4%)	83 (100.0%)	206 (98.6%)
Yes	2 (1.9%)	0 (0.0%)	1 (5.6%)	0 (0.0%)	3 (1.4%)
**Congestive heart failure**					
No	88 (83.8%)	3 (100.0%)	16 (88.9%)	77 (92.8%)	184 (88.0%)
Yes	17 (16.2%)	0 (0.0%)	2 (11.1%)	6 (7.2%)	25 (12.0%)
**No complications**					
No	77 (73.3%)	2 (66.7%)	9 (50.0%)	48 (57.8%)	136 (65.1%)
Yes	28 (26.7%)	1 (33.3%)	9 (50.0%)	35 (42.2%)	73 (34.9%)

Supplemental Table 1 (A more detailed version of table 1). A retrospective analysis of critically ill patients admitted to the intensive care unit at North Bristol NHS Trust (UK) with a positive PCR result for SARS-CoV-2 and for whom ABO blood group data was available.

	ABO Blood groups					Secretor phenotype		
	A (N=55)	AB (N=7)	B (N=18)	O (N=47)	Total (N=127)	Non-Secretor (N=21)	Secretor (N=106)	Total (N=127)
**ABO**								
A						5 (23.8%)	50 (47.2%)	55 (43.3%)
AB						2 (9.5%)	5 (4.7%)	7 (5.5%)
B						3 (14.3%)	15 (14.2%)	18 (14.2%)
O						11 (52.4%)	36 (34.0%)	47 (37.0%)
**PCR Secretor**								
Non-Secretor	5 (9.1%)	2 (28.6%)	3 (16.7%)	11 (23.4%)	21 (16.5%)			
Secretor	50 (90.9%)	5 (71.4%)	15 (83.3%)	36 (76.6%)	106 (83.5%)			
**Age**	61.1 ± 18.2	66.0 ± 17.3	51.4 ± 10.5	59.4 ± 14.0	59.4 ± 16.0	58.5 ± 13.1	59.6 ± 16.5	59.4 ± 16.0
**Sex**								
Female	26 (47.3%)	2 (28.6%)	7 (38.9%)	18 (38.3%)	53 (41.7%)	12 (57.1%)	41 (38.7%)	53 (41.7%)
Male	29 (52.7%)	5 (71.4%)	11 (61.1%)	29 (61.7%)	74 (58.3%)	9 (42.9%)	65 (61.3%)	74 (58.3%)
**Caucasian**								
No	8 (14.5%)	1 (14.3%)	5 (27.8%)	6 (12.8%)	20 (15.7%)	5 (23.8%)	15 (14.2%)	20 (15.7%)
Unknown	3 (5.5%)	3 (42.9%)	2 (11.1%)	3 (6.4%)	11 (8.7%)	4 (19.0%)	7 (6.6%)	11 (8.7%)
Yes	44 (80.0%)	3 (42.9%)	11 (61.1%)	38 (80.9%)	96 (75.6%)	12 (57.1%)	84 (79.2%)	96 (75.6%)
**Systolic BP**	134.6 ± 21.5	128.1 ± 15.8	132.1 ± 29.8	128.5 ± 16.3	131.6 ± 20.9	125.4 ± 18.5	132.9 ± 21.2	131.6 ± 20.9
**Diastolic BP**	73.7 ± 14.3	78.6 ± 5.4	82.2 ± 14.3	74.2 ± 13.7	75.4 ± 13.9	74.6 ± 13.7	75.5 ± 14.0	75.4 ± 13.9
**Heart rate bpm**	90.7 ± 23.9	83.7 ± 14.2	102.4 ± 22.4	91.6 ± 17.5	92.3 ± 21.3	91.4 ± 19.0	92.5 ± 21.8	92.3 ± 21.3
**Temperature C**	37.6 ± 0.9	37.1 ± 1.1	37.6 ± 0.8	37.6 ± 0.9	37.6 ± 0.9	37.8 ± 0.7	37.5 ± 0.9	37.6 ± 0.9
**Respiratory Rate per min**	21.7 ± 4.1	22.7 ± 8.0	24.8 ± 6.8	21.6 ± 5.3	22.1 ± 5.3	21.6 ± 4.8	22.3 ± 5.4	22.1 ± 5.3
**Haemoglobin g/L**	134.4 ± 17.8	132.7 ± 16.0	144.6 ± 17.7	133.7 ± 20.2	135.5 ± 18.8	137.5 ± 17.1	135.1 ± 19.1	135.5 ± 18.8
**White cell count x10e9/L**	9.1 ± 4.8	8.0 ± 2.2	8.7 ± 4.0	8.1 ± 5.1	8.6 ± 4.7	7.4 ± 2.2	8.8 ± 5.0	8.6 ± 4.7
**Neutrophil count x10e9/L**	7.2 ± 4.6	6.6 ± 2.1	6.7 ± 3.3	7.4 ± 8.9	7.1 ± 6.3	5.6 ± 2.3	7.5 ± 6.8	7.1 ± 6.3
**Lymphocyte count x10e9/L**	1.1 ± 0.6	0.9 ± 0.3	1.2 ± 0.5	1.2 ± 0.7	1.1 ± 0.6	1.2 ± 0.5	1.1 ± 0.7	1.1 ± 0.6
**Platelet count x10e9/L**	245.7 ± 107.4	253.7 ± 72.8	247.7 ± 78.4	248.6 ± 120.0	247.5 ± 106.3	230.6 ± 77.7	250.8 ± 111.1	247.5 ± 106.3
**Sodium mmol/L**	137.4 ± 3.7	136.6 ± 2.2	129.6 ± 31.5	136.5 ± 3.1	135.9 ± 12.2	137.3 ± 2.9	135.6 ± 13.3	135.9 ± 12.2
**Urea mmol/L**	6.9 ± 5.3	6.0 ± 3.6	4.8 ± 2.0	6.5 ± 3.8	6.4 ± 4.3	5.3 ± 2.6	6.6 ± 4.6	6.4 ± 4.3
**eGFR ml/min/1.73m2**	71.3 ± 24.4	75.1 ± 14.2	79.8 ± 12.0	70.8 ± 23.6	72.5 ± 22.3	75.2 ± 20.4	72.0 ± 22.7	72.5 ± 22.3
**Albumin g/L**	32.1 ± 5.2	30.4 ± 3.4	35.1 ± 2.9	32.4 ± 4.5	32.5 ± 4.7	31.0 ± 4.0	32.8 ± 4.7	32.5 ± 4.7
**Ferritin µg/L**	712.8 ± 780.2	2262.8 ± 1537.1	623.8 ± 648.5	910.3 ± 1051.7	833.8 ± 945.4	976.8 ± 1114.4	809.4 ± 918.7	833.8 ± 945.4
**pro BNP pg/ml**	2753.9 ± 8546.9	86.1 ± 65.0	178.1 ± 364.6	1915.0 ± 5670.2	1991.4 ± 6754.2	299.2 ± 530.2	2283.2 ± 7276.3	1991.4 ± 6754.2
**TNT pg/ml**	34.7 ± 117.5	10.3 ± 4.0	10.0 ± 8.1	53.9 ± 142.6	37.3 ± 117.4	14.6 ± 13.1	41.3 ± 126.7	37.3 ± 117.4
**KL 6 U/ml**	492.5 ± 574.8	315.8 ± 192.9	378.3 ± 213.8	407.5 ± 337.4	431.1 ± 429.9	412.4 ± 265.4	435.5 ± 461.6	431.1 ± 429.9
**IL 6 pg/ml**	95.3 ± 166.6	70.4 ± 65.1	111.9 ± 176.8	76.4 ± 91.7	89.4 ± 139.8	95.8 ± 112.2	88.1 ± 145.4	89.4 ± 139.8
**Procalcitonin ng/ml**	2.6 ± 14.7	0.1 ± 0.0	0.1 ± 0.1	7.7 ± 37.8	4.0 ± 24.9	0.3 ± 0.8	4.6 ± 27.0	4.0 ± 24.9
**LDH U/L**	611.3 ± 218.1	788.2 ± 185.3	678.2 ± 278.2	607.5 ± 262.2	626.2 ± 242.7	730.3 ± 166.3	608.0 ± 250.0	626.2 ± 242.7
**suPAR ng/ml**	6.7 ± 4.5	5.7 ± 3.5	5.7 ± 3.6	6.0 ± 3.0	6.3 ± 3.8	5.5 ± 1.5	6.4 ± 4.0	6.3 ± 3.8

Supplemental Table 2 - Retrospective analysis of patients admitted to North Bristol NHS Trust (UK) and enrolled onto the DISCOVER study for which ABO phenotype and full clinical measurements data was available. All clinical measurement were taken at the time of hospital admission.

	ABO blood group A	ABO blood group O	ABO blood group Total	Risk ratio (95% CI)	P-value†
**Number of patients / Percentage of total**	152 (55.27%)	123 (44.73%)	275 (100%)	1.24 (1.05, 1.47)	**0.0111‡**
**Age (± SD, years)**	69.2 ± 19.1	67.4 ± 18.5	68.4 ± 18.8	------------	0.2962
**Gender**				1.28 (1.02, 1.61)	**0.0363**
Female	60 (39.5%)	65 (52.8%)	125 (45.5%)		
Male	92 (60.5%)	58 (47.2%)	150 (54.5%)		
**Inpatient death**				1.00 (0.64, 1.54)	0.6582
Unknown/No data	2 (1.3%)	0 (0.0%)	2 (0.7%)		
No	116 (76.3%)	95 (77.2%)	211 (76.7%)		
Yes	34 (22.4%)	28 (22.8%)	62 (22.5%)		
**Acute renal failure**				1.16 (0.74, 1.82)	0.6026
No	116 (76.3%)	98 (79.7%)	214 (77.8%)		
Yes	36 (23.7%)	25 (20.3%)	61 (22.2%)		
**Liver dysfunction**				1.10 (0.57, 2.08)	0.9227
No	133 (87.5%)	109 (88.6%)	242 (88.0%)		
Yes	19 (12.5%)	14 (11.4%)	33 (12.0%)		
**Cardiovascular complication***				2.56 (1.43, 4.55)	**0.0011**
No	111 (73.0%)	110 (89.4%)	221 (80.4%)		
Yes	41 (27.0%)	13 (10.6%)	54 (19.6%)		

*Encompasses Non-ST-elevation myocardial infarction (NSTEMI), ST-elevation myocardial infarction (STEMI), Atrial fibrillation, stroke, brain haemorrhage, deep vein thrombus (DVT), pulmonary embolus (PE) and congestive heart failure.

†Statistical comparisons for discrete variables were performed with Fisher's Exact Test, and statistical comparisons for continuous variables were performed with the Wilcoxon rank sum test. Blood group O is used as the baseline for comparison.

‡Compared against the official statistics provided by NHS Blood and Transplant, comprising blood group distribution in the United Kingdom. Comparison performed with Pearson's chi square frequency test.

Supplemental Table 3 – Statistical analysis of critically ill patients admitted to the intensive care in North Bristol NHS Trust (UK) with a positive PCR result for SARS-CoV-2 and that for whom ABO blood group data was available (Table 1) combined with data obtained through the DISCOVER study (Table 3).

	ABO blood group A	ABO blood group O	ABO blood group Total	Risk ratio (95% CI)	P-value†
**Cardiovascular complication*, male**				2.50 (1.18, 5.26)	**0.0168**
No	64 (69.6%)	51 (87.9%)	115 (76.7%)		
Yes	28 (30.4%)	7 (12.1%)	35 (23.3%)		
**Cardiovascular complication*, female**				2.33 (0.95, 5.88)	**0.0919**
No	47 (78.3%)	59 (90.8%)	106 (84.8%)		
Yes	13 (21.7%)	6 (9.2%)	19 (15.2%)		

*Encompasses Non-ST-elevation myocardial infarction (NSTEMI), ST-elevation myocardial infarction (STEMI), Atrial fibrillation, stroke, brain haemorrhage, deep vein thrombus (DVT), pulmonary embolus (PE) and congestive heart failure.

†Statistical comparisons for discrete variables were performed with Fisher's Exact Test, and statistical comparisons for continuous variables were performed with the Wilcoxon rank sum test. Blood group O is used as the baseline for comparison.

Supplemental Table 4 – Statistical analysis of patients with cardiovascular complications, split into male and female, from Supplemental Table 3.
